# Supplementary material for: Higher mindfulness and lower self-absorption predict greater compassion within romantic relationships
Source: Commun Psychol. 2026 Jun 6;4:91. doi: 10.1038/s44271-026-00483-y (PMC13242517; doi:10.1038/s44271-026-00483-y)
Supplement: Supplementary file 2 — Supplementary Information [file 44271_2026_483_MOESM2_ESM.pdf]

Supplementary Information

Supplementary Note 1: Daily Diary Results

Model Selection

Table S1a

Model Selection for Hypothesis 1: Partner Ratings of Compassion

Model	df	AIC	BIC	logLik	Test	L.Ratio	p-value
APIM-Distinguishable	26	5468.164	5601.630	-2708.082	—	—	—
**APIM-Indistinguishable**	**18**	**5456.516**	**5548.916**	**-2710.258**	**1 vs 2**	**4.35235**	**0.824**
**Non-APIM**	11	5541.127	5597.594	-2759.564	2 vs 3	98.61115	<0.0001

Table S1b

Model Selection for Hypothesis 1: Self Ratings of Compassion

Model	df	AIC	BIC	logLik	Test	L.Ratio	p-value
APIM-Distinguishable	26	5188.071	5321.516	-2568.036	—	—	—
**APIM-Indistinguishable**	**18**	**5177.666**	**5270.051**	**-2570.833**	**1 vs 2**	**5.59425**	**0.6926**
**Non-APIM**	11	5213.755	5270.213	-2595.878	2 vs 3	50.08990	<0.0001

Table S1c

Model Selection for Hypothesis 2: Path a

Model	df	AIC	BIC	logLik	Test	L.Ratio	p-value
APIM-Distinguishable	26	8918.049	9069.918	-4433.025	—	—	—
**APIM-Indistinguishable**	**18**	**8908.503**	**9013.642**	**-4436.251**	**1 vs 2**	**6.45348**	**0.5966**
**Non-APIM**	11	8939.987	9004.239	-4458.993	2 vs 3	45.48402	<0.0001

Table S1d

Model Selection for Hypothesis 2: Full Model, Partner Ratings of Compassion

Model	df	AIC	BIC	logLik	Test	L.Ratio	p-value
**APIM-Distinguishable**	38	5406.925	5601.990	-2665.463	—	—	—
**APIM-Indistinguishable**	**20**	**5403.388**	**5536.853**	**-2675.694**	**1 vs 2**	**19.82224**	**0.0705**
**Non-APIM**	12	5492.185	5569.184	-2731.092	2 vs 3	110.79700	<0.0001

Table S1e

Model Selection for Hypothesis 2: Full Model, Self Ratings of Compassion.

Model	df	AIC	BIC	logLik	Test	L.Ratio	p-value
**APIM-Distinguishable**	38	5153.413	5348.448	-2538.706	—	—	—
**APIM-Indistinguishable**	**26**	**5147.535**	**5280.980**	**-2547.768**	**1 vs 2**	**18.12212**	**0.112**
**Non-APIM**	15	5179.120	5256.108	-2574.560	2 vs 3	53.58513	<0.0001

Table S1f

Model Selection for Hypothesis 3: Partner Ratings of Compassion

Model	df	AIC	BIC	logLik	Test	L.Ratio	p-value
**APIM-Distinguishable**	50	5341.757	5598.422	-2620.879	—	—	—
**APIM-Indistinguishable**	**38**	**5335.571**	**5530.636**	**-2629.785**	**1 vs 2**	**28.88053**	**0.0901**
**Non-APIM**	21	5463.438	5571.237	-2710.719	2 vs 3	161.86646	<0.0001

Table S1g

Model Selection for Hypothesis 3: Self Ratings of Compassion

Model	df	AIC	BIC	logLik	Test	L.Ratio	p-value
**APIM-Distinguishable**	58	5021.655	5319.339	-2452.827	—	—	—
**APIM-Indistinguishable**	**38**	**5005.710**	**5200.745**	**-2464.855**	**1 vs 2**	**24.05518**	**0.24**

**Non-APIM**	21	5033.575	5141.357	-2495.787	2 vs 3	61.86484	<0.0001

Model Output

Table S2a

Hypothesis 1: Partner Ratings of Compassion

Predictor	Estimate	SE	95% CI lower	95% CI upper	df	t	p
**Intercept**	4.25	1.16	1.97	6.52	1048	3.66	<.001
**State mindfulness - acceptance (actor)**	0.19	0.06	0.07	0.31	1048	3.10	0.002
**State mindfulness - attention (actor)**	0.05	0.06	-0.08	0.18	1048	0.80	0.425
**Trait mindfulness -acceptance (actor)**	-0.06	0.15	-0.36	0.24	88	-0.40	0.687
**Trait mindfulness -attention (actor)**	0.52	0.16	0.19	0.85	88	3.15	0.002
**State mindfulness - acceptance (partner)**	0.31	0.06	0.17	0.44	1048	4.53	<.001
**State mindfulness - attention (partner)**	-0.09	0.06	-0.21	0.03	1048	-1.53	0.127
**Trait mindfulness - acceptance (partner)**	-0.12	0.16	-0.44	0.20	88	-0.77	0.443

**Trait mindfulness – attention (partner)**	0.48	0.17	0.15	0.81	88	2.86	0.005

Random Effect	Grouping Level	Estimated SD of the random effect
**Intercept**	Dyad	1.25
**Intercept**	Participant (within dyad)	1.29
**State mindful acceptance (actor)**	Participant	0.34
**State mindful attention (actor)**	Participant	0.37
**State mindful acceptance (partner)**	Participant	0.53
**State mindful attention (partner)**	Participant	0.33
**Residual**	—	1.66

Note: p values reported exactly to three decimals; p < .001 when smaller. Actor = participant's own state/trait; Partner = partner's concurrent state/trait.

Table S2b

Hypothesis 1: Self Ratings of Compassion

Predictor	Estimate	SE	95% CI lower	95% CI upper	df	t	p
**Intercept**	4.91	0.90	3.14	6.68	1043	5.44	<.001
**State mindfulness — acceptance (actor)**	0.27	0.06	0.16	0.39	1043	4.66	<.001
**State mindfulness — attention (actor)**	0.07	0.06	-0.04	0.19	1043	1.31	0.190
**Trait mindfulness -acceptance (actor)**	0.26	0.13	0.01	0.52	91	2.06	0.042
**Trait mindfulness**	0.52	0.14	0.25	0.79	91	3.90	<.001

**-attention (actor)**							
**State mindfulness — acceptance (partner)**	-0.02	0.06	-0.15	0.11	1043	-0.28	0.778
**State mindfulness — attention (partner)**	-0.03	0.06	-0.15	0.08	1043	-0.60	0.549
**Trait mindfulness - acceptance (partner)**	-0.30	0.12	-0.55	-0.06	91	-2.46	0.016
**Trait mindfulness – attention (partner)**	0.29	0.13	0.03	0.55	91	2.20	0.030

Random Effect	Grouping Level	Estimated SD of the random effect
**Intercept**	Dyad	0.95
**Intercept**	Participant (within dyad)	1.06
**State mindful acceptance (actor)**	Participant	0.39
**State mindful attention (actor)**	Participant	0.33
**State mindful acceptance (partner)**	Participant	0.52
**State mindful attention (partner)**	Participant	0.28
**Residual**	—	1.50

Note: p values reported exactly to three decimals; p < .001 when smaller. Actor = participant's own state/trait; Partner = partner's concurrent state/trait.

Table S3a

Hypothesis 2: Partner Ratings of Compassion

Outcome	Predictor	Estimate	SE	95% CI lower	95% CI upper	df	t	p
**Compassion**	Intercept	7.44	1.17	4.74	9.96	6070	6.36	<.001

**Self-absorption (actor)**	Intercept	9.17	0.48	8.17	10.04	6070	19.20	<.001
**Self-absorption (partner)**	Intercept	8.25	0.51	7.27	9.48	6070	16.03	<.001
**Compassion**	Self-absorption (actor)	-0.22	0.04	-0.32	-0.13	6070	-4.97	<.001
**Compassion**	Self-absorption (partner)	-0.11	0.04	-0.19	-0.02	6070	-2.43	0.015
**Compassion**	State mindfulness — acceptance (actor)	0.07	0.06	-0.05	0.20	6070	1.19	0.234
**Compassion**	State mindfulness — attention (actor)	-0.003	0.06	-0.13	0.14	6070	-0.05	0.963
**Compassion**	State mindfulness — acceptance (partner)	0.26	0.07	0.12	0.40	6070	3.86	<.001
**Compassion**	State mindfulness — attention (partner)	-0.12	0.06	-0.25	0.00	6070	-2.06	0.039
**Compassion**	Trait mindfulness — acceptance (actor)	-0.11	0.16	-0.45	0.20	6070	-0.66	0.512
**Compassion**	Trait mindfulness — attention (actor)	0.24	0.18	-0.07	0.63	6070	1.32	0.188
**Compassion**	Trait mindfulness — acceptance (partner)	0.06	0.17	-0.44	0.39	6070	0.33	0.742
**Compassion**	Trait mindfulness — attention (partner)	0.32	0.18	-0.02	0.74	6070	1.76	0.079
**Self-absorption (actor)**	State mindfulness — acceptance (actor)	-0.24	0.04	-0.32	-0.16	6070	-6.60	<.001
**Self-absorption (actor)**	State mindfulness — attention (actor)	-0.31	0.04	-0.39	-0.23	6070	-8.90	<.001

**Self-absorption (actor)**	State mindfulness — acceptance (partner)	-0.10	0.03	-0.16	-0.04	6070	-3.65	<.001
**Self-absorption (actor)**	State mindfulness — attention (partner)	-0.02	0.03	-0.07	0.04	6070	-0.79	0.430
**Self-absorption (actor)**	Trait mindfulness — acceptance (actor)	-0.14	0.06	-0.29	-0.01	6070	-2.18	0.029
**Self-absorption (actor)**	Trait mindfulness — attention (actor)	-0.92	0.07	-1.07	-0.76	6070	-12.82	<.001
**Self-absorption (actor)**	Trait mindfulness — acceptance (partner)	-0.01	0.06	-0.15	0.11	6070	-0.13	0.895
**Self-absorption (actor)**	Trait mindfulness — attention (partner)	-0.10	0.07	-0.26	0.07	6070	-1.38	0.166
**Self-absorption (actor)**	Self-absorption (partner)	0.12	0.02	0.03	0.19	6070	6.07	<.001
**Self-absorption (partner)**	State mindfulness — acceptance (actor)	-0.08	0.03	-0.14	-0.02	6070	-2.83	0.005
**Self-absorption (partner)**	State mindfulness — attention (actor)	-0.001	0.03	-0.05	0.05	6070	-0.02	0.984
**Self-absorption (partner)**	State mindfulness — acceptance (partner)	-0.23	0.04	-0.30	-0.15	6070	-6.26	<.001
**Self-absorption (partner)**	State mindfulness — attention (partner)	-0.29	0.04	-0.37	-0.22	6070	-8.02	<.001
**Self-absorption (partner)**	Trait mindfulness — acceptance (actor)	0.03	0.07	-0.12	0.15	6070	0.38	0.702
**Self-absorption (partner)**	Trait mindfulness — attention (actor)	0.01	0.08	-0.18	0.20	6070	0.12	0.902

**Self-absorption (partner)**	Trait mindfulness — acceptance (partner)	-0.15	0.07	-0.30	-0.01	6070	-2.31	0.021
**Self-absorption (partner)**	Trait mindfulness — attention (partner)	-0.94	0.07	-1.11	-0.79	6070	-12.96	<.001

Random effect	Grouping level	Outcome block	Estimated SD of the random effect
**Intercept**	Dyad	—	0.50
**Intercept**	Participant (within dyad)	Compassion	1.54
**Intercept**	Participant (within dyad)	Self-absorption (actor)	4.34
**Intercept**	Participant (within dyad)	Self-absorption (partner)	3.96
**State acceptance (actor)**	Participant	Compassion	10.42
**State attention (actor)**	Participant	Compassion	7.60
**State acceptance (partner)**	Participant	Compassion	4.86
**State attention (partner)**	Participant	Compassion	8.73
**Self-absorption (actor)**	Participant	Compassion	16.74
**Self-absorption (partner)**	Participant	Compassion	12.96
**State acceptance (actor)**	Participant	Self-absorption (actor)	7.22
**State attention (actor)**	Participant	Self-absorption (actor)	8.58
**State acceptance (partner)**	Participant	Self-absorption (actor)	13.78
**State attention (partner)**	Participant	Self-absorption (actor)	22.18
**Self-absorption (partner)**	Participant	Self-absorption (actor)	20.78
**State acceptance (actor)**	Participant	Self-absorption (partner)	13.12
**State attention (actor)**	Participant	Self-absorption (partner)	24.44
**State acceptance (partner)**	Participant	Self-absorption (partner)	7.28
**State attention (partner)**	Participant	Self-absorption (partner)	7.72
**Residual**	—	—	1.59

Note: p values reported exactly to three decimals; p < .001 when smaller. Actor = participant's own state/trait; Partner = partner's concurrent state/trait.

Figure S1

Indirect Effects of Actor Mindfulness on Partner Ratings of Compassion via Self–Absorption, Controlling for Partner Effects

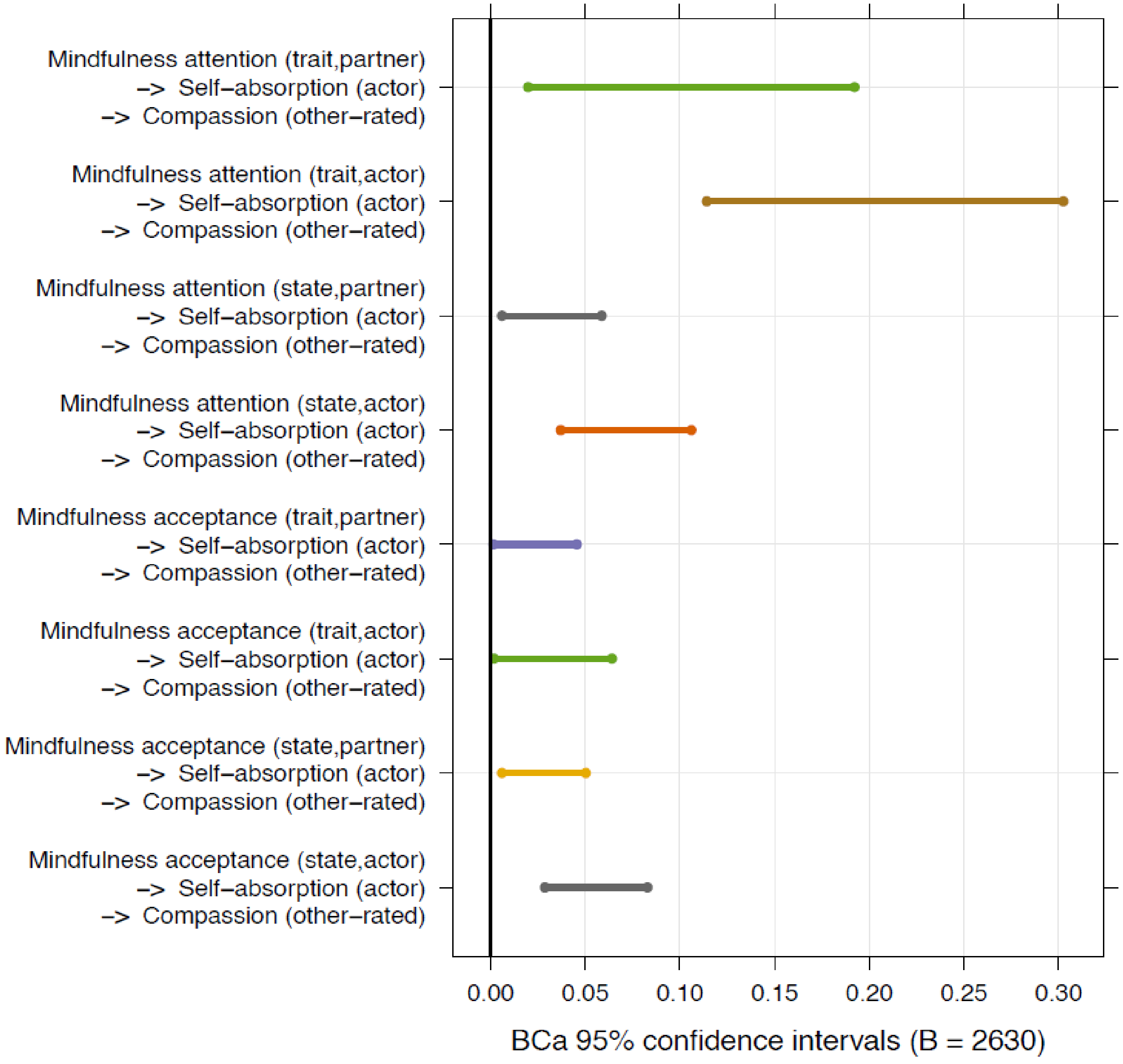

Note. N=225 participants

Table S3b

Hypothesis 2: Self Ratings of Compassion

**Outcome**	**Predictor**	**Estimate**	**SE**	**95% CI lower**	**95% CI upper**	**df**	**t**	**p**
**Compassion**	Intercept	7.06	0.95	5.00	8.98	6066	7.44	<.001
**Self-absorption (actor)**	Intercept	9.15	0.48	8.12	10.04	6066	19.21	<.001
**Self-absorption (partner)**	Intercept	8.26	0.51	7.25	9.43	6066	16.07	<.001
**Compassion**	Self-absorption (actor)	-0.21	0.04	-0.28	-0.14	6066	-5.84	<.001
**Compassion**	Self-absorption (partner)	-0.04	0.04	-0.11	0.04	6066	-1.13	0.259
**Compassion**	State mindfulness — acceptance (actor)	0.21	0.06	0.08	0.32	6066	3.67	<.001
**Compassion**	State mindfulness — attention (actor)	-0.01	0.06	-0.12	0.10	6066	-0.20	0.845
**Compassion**	State mindfulness — acceptance (partner)	-0.07	0.06	-0.19	0.06	6066	-1.02	0.307
**Compassion**	State mindfulness — attention (partner)	-0.05	0.06	-0.18	0.08	6066	-0.90	0.367
**Compassion**	Trait mindfulness — acceptance (actor)	0.22	0.14	-0.06	0.52	6066	1.65	0.100
**Compassion**	Trait mindfulness — attention (actor)	0.35	0.15	0.05	0.65	6066	2.35	0.019
**Compassion**	Trait mindfulness — acceptance (partner)	-0.29	0.13	-0.53	-0.03	6066	-2.15	0.031
**Compassion**	Trait mindfulness — attention (partner)	0.22	0.14	-0.02	0.45	6066	1.54	0.124
**Self-absorption (actor)**	State mindfulness — acceptance (actor)	-0.24	0.04	-0.32	-0.16	6066	-6.61	<.001

**Self-absorption (actor)**	State mindfulness — attention (actor)	-0.31	0.04	-0.39	-0.23	6066	-8.93	<.001
**Self-absorption (actor)**	State mindfulness — acceptance (partner)	-0.10	0.03	-0.16	-0.04	6066	-3.61	<.001
**Self-absorption (actor)**	State mindfulness — attention (partner)	-0.02	0.03	-0.07	0.04	6066	-0.77	0.440
**Self-absorption (actor)**	Trait mindfulness — acceptance (actor)	-0.14	0.06	-0.29	0.00	6066	-2.16	0.031
**Self-absorption (actor)**	Trait mindfulness — attention (actor)	-0.92	0.07	-1.08	-0.76	6066	-12.82	<.001
**Self-absorption (actor)**	Trait mindfulness — acceptance (partner)	-0.01	0.06	-0.15	0.11	6066	-0.12	0.904
**Self-absorption (actor)**	Trait mindfulness — attention (partner)	-0.10	0.07	-0.26	0.08	6066	-1.37	0.172
**Self-absorption (actor)**	Self-absorption (partner)	0.12	0.02	0.03	0.19	6066	6.11	<.001
**Self-absorption (partner)**	State mindfulness — acceptance (actor)	-0.08	0.03	-0.14	-0.03	6066	-2.82	0.005
**Self-absorption (partner)**	State mindfulness — attention (actor)	0.00	0.03	-0.05	0.06	6066	-0.01	0.990
**Self-absorption (partner)**	State mindfulness — acceptance (partner)	-0.23	0.04	-0.30	-0.16	6066	-6.26	<.001
**Self-absorption (partner)**	State mindfulness — attention (partner)	-0.29	0.04	-0.37	-0.22	6066	-8.02	<.001
**Self-absorption (partner)**	Trait mindfulness — acceptance (actor)	0.03	0.07	-0.12	0.14	6066	0.38	0.703

**Self-absorption (partner)**	Trait mindfulness — attention (actor)	0.01	0.08	-0.17	0.20	6066	0.13	0.898
**Self-absorption (partner)**	Trait mindfulness — acceptance (partner)	-0.15	0.07	-0.30	-0.01	6066	-2.31	0.021
**Self-absorption (partner)**	Trait mindfulness — attention (partner)	-0.94	0.07	-1.12	-0.78	6066	-12.99	<.001

**Random effect**	**Grouping level**	**Outcome block**	**Estimated SD of the random effect**
**Intercept**	Dyad	—	0.49
**Intercept**	Participant (within dyad)	Compassion	2.28
**Intercept**	Participant (within dyad)	Self-absorption (actor)	3.74
**Intercept**	Participant (within dyad)	Self-absorption (partner)	3.41
**State acceptance (actor)**	Participant	Compassion	6.64
**State attention (actor)**	Participant	Compassion	7.70
**State acceptance (partner)**	Participant	Compassion	4.63
**State attention (partner)**	Participant	Compassion	9.16
**Self-absorption (actor)**	Participant	Compassion	30.73
**Self-absorption (partner)**	Participant	Compassion	19.51
**State acceptance (actor)**	Participant	Self-absorption (actor)	6.26
**State attention (actor)**	Participant	Self-absorption (actor)	7.48
**State acceptance (partner)**	Participant	Self-absorption (actor)	11.91
**State attention (partner)**	Participant	Self-absorption (actor)	18.82
**Self-absorption (partner)**	Participant	Self-absorption (actor)	17.58
**State acceptance (actor)**	Participant	Self-absorption (partner)	11.38
**State attention (actor)**	Participant	Self-absorption (partner)	20.97

**State acceptance (partner)**	Participant	Self-absorption (partner)	6.32
**State attention (partner)**	Participant	Self-absorption (partner)	6.71
**Residual**	—	—	1.48

Note: p values reported exactly to three decimals; p < .001 when smaller. Actor = participant's own state/trait; Partner = partner's concurrent state/trait.

Figure S2

Indirect Effects of Actor Mindfulness on Self Ratings of Compassion via Self–absorption, Controlling for Partner Effects

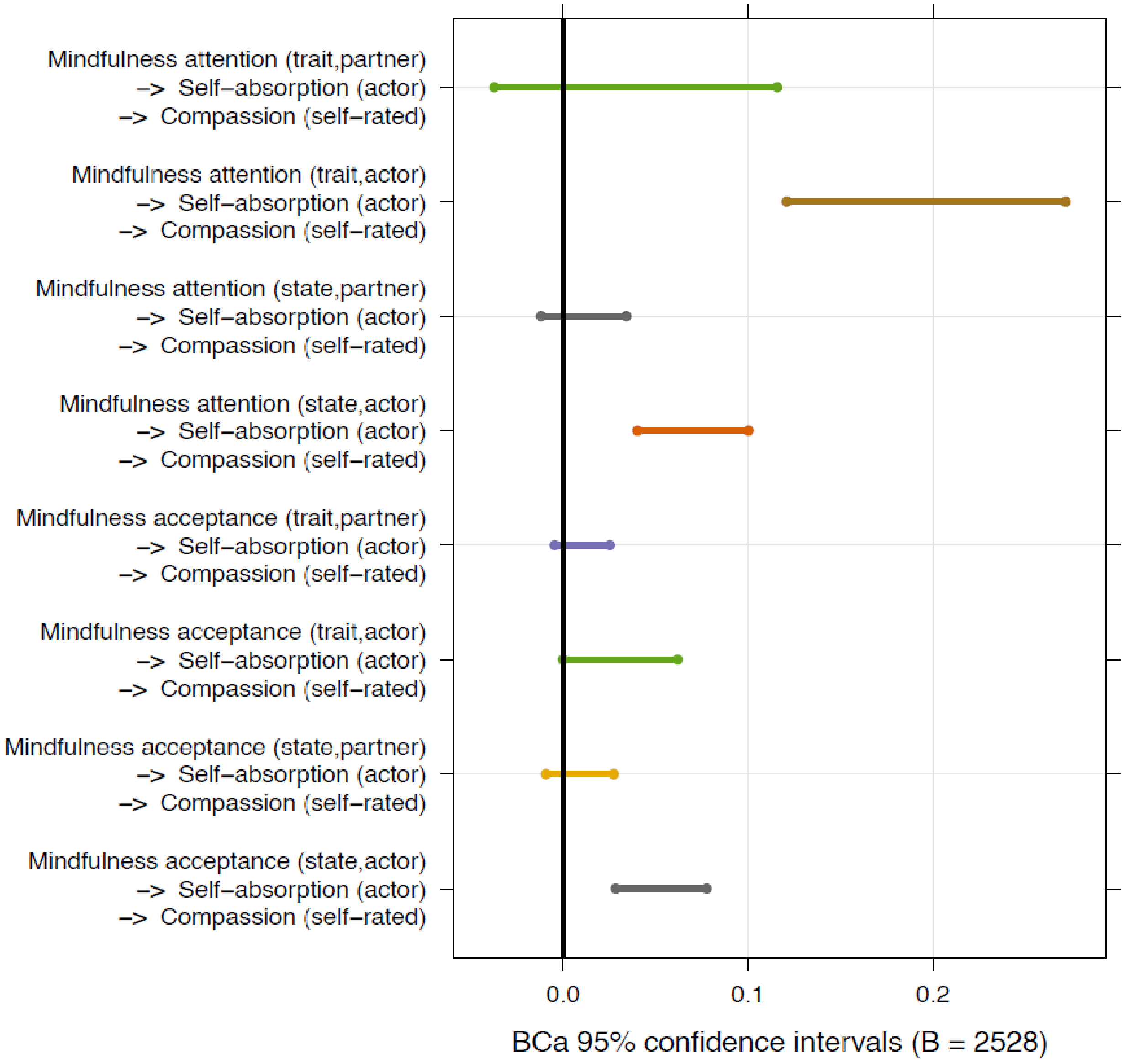

Note. N=225 participants

Table S4a

Hypothesis 3: Partner Ratings of Compassion

Predictor	Estimate	SE	95% CI lower	95% CI upper	df	t	p
**Intercept**	3.35	3.1	-2.73	9.43	1042	1.08	0.28
**State mindfulness — acceptance (actor)**	0.08	0.06	-0.04	0.2	1042	1.3	0.193
**State mindfulness — attention (actor)**	0.04	0.06	-0.08	0.15	1042	0.61	0.541
**Trait mindfulness - acceptance (actor)**	-0.04	0.14	-0.33	0.24	82	-0.29	0.771
**Trait mindfulness - attention (actor)**	0.16	0.19	-0.23	0.54	82	0.82	0.415
**State mindfulness — acceptance (partner)**	0.24	0.07	0.1	0.37	1042	3.48	<.001
**State mindfulness — attention (partner)**	-0.11	0.06	-0.22	0	1042	-1.93	0.054
**Trait mindfulness - acceptance (partner)**	-0.22	0.15	-0.52	0.09	82	-1.43	0.157
**Trait mindfulness – attention (partner)**	0	0.2	-0.39	0.39	82	-0.01	0.994
**State self-absorption (actor)**	-0.13	0.05	-0.22	-0.03	1042	-2.48	0.013
**State self-construal (actor)**	0.13	0.04	0.05	0.22	1042	3.1	0.002
**Trait self-absorption (actor)**	0.01	0.44	-0.86	0.88	82	0.03	0.978
**Trait self-construal (actor)**	0.23	0.18	-0.13	0.6	82	1.27	0.208
**State self-absorption (partner)**	-0.04	0.04	-0.12	0.05	1042	-0.91	0.364

**State self-construal (partner)**	0.16	0.05	0.06	0.26	1042	3.11	0.002
**Trait self-absorption (partner)**	0.47	0.44	-0.41	1.35	82	1.07	0.289
**Trait self-construal (partner)**	0.6	0.19	0.23	0.98	82	3.19	0.002
**State SA × State SC (actor)**	-0.02	0.02	-0.06	0.02	1042	-1.16	0.246
**Trait SA × Trait SC (actor)**	-0.02	0.05	-0.11	0.07	82	-0.41	0.680
**State SA × State SC (partner)**	0.01	0.02	-0.03	0.04	1042	0.42	0.671
**Trait SA × Trait SC (partner)**	-0.1	0.05	-0.2	0	82	-2.09	0.040

Random effect	Grouping level	Estimated SD of the random effect
**Intercept**	Dyad	0.87
**Intercept**	Participant (within dyad)	1.37
**State mindfulness—acceptance (actor)**	Participant	0.34
**State mindfulness—attention (actor)**	Participant	0.24
**State SA (actor)**	Participant (within dyad)	0.27
**State SC (actor)**	Participant	0.20
**State mindfulness—acceptance (partner)**	Participant	0.55
**State mindfulness—attention (partner)**	Participant	0.29
**State SA (partner)**	Participant	0.20
**State SC (partner)**	Participant	0.34
**Residual**	—	1.44

Note: p values reported exactly to three decimals; $p < .001$ when smaller. Actor = participant's own state/trait; Partner = partner's concurrent state/trait.

Table S4b

Hypothesis 3: Self Ratings of Compassion

Predictor	Estimate	SE	95% CI lower	95% CI upper	df	t	p

**Intercept**	5.38	2.34	0.78	9.97	1037	2.29	0.022
**State mindfulness — acceptance (actor)**	0.11	0.06	-0.01	0.22	1037	1.84	0.066
**State mindfulness — attention (actor)**	0.02	0.06	-0.1	0.13	1037	0.28	0.783
**Trait mindfulness - acceptance (actor)**	0.23	0.12	-.004	0.46	85	1.95	0.054
**Trait mindfulness - attention (actor)**	0.27	0.15	-0.02	0.56	85	1.83	0.071
**State mindfulness — acceptance (partner)**	-0.02	0.06	-0.14	0.09	1037	-0.39	0.695
**State mindfulness — attention (partner)**	0	0.06	-0.11	0.11	1037	-0.03	0.980
**Trait mindfulness - acceptance (partner)**	-0.2	0.11	-0.43	0.02	85	-1.83	0.071
**Trait mindfulness – attention (partner)**	0.09	0.15	-0.21	0.38	85	0.58	0.562
**State self-absorption (actor)**	-0.13	0.04	-0.21	-0.05	1037	-3.16	0.002
**State self-construal (actor)**	0.33	0.04	0.25	0.41	1037	8.46	<.001
**Trait self-absorption (actor)**	-0.57	0.34	-1.24	0.1	85	-1.7	0.092
**Trait self-construal (actor)**	0.17	0.14	-0.12	0.45	85	1.17	0.244
**State self-absorption (partner)**	0.05	0.04	-0.03	0.13	1037	1.15	0.249

**State self-construal (partner)**	0.03	0.04	-0.05	0.11	1037	0.74	0.459
**Trait self-absorption (partner)**	-0.1	0.32	-0.73	0.54	85	-0.31	0.759
**Trait self-construal (partner)**	0.05	0.14	-0.22	0.32	85	0.39	0.697
**State SA × State SC (actor)**	0.03	0.02	0	0.07	1037	2.01	0.044
**Trait SA × Trait SC (actor)**	0.05	0.04	-0.02	0.13	85	1.51	0.136
**State SA × State SC (partner)**	-0.05	0.02	-0.08	-0.01	1037	-2.74	0.006
**Trait SA × Trait SC (partner)**	0.01	0.03	-0.06	0.08	85	0.18	0.857

**Random effect**	**Grouping level**	**Estimated SD of the random effect**
**Intercept**	Dyad	0.70
**Intercept**	Participant (within dyad)	0.94
**State mindfulness—acceptance (actor)**	Participant	0.39
**State mindfulness—attention (actor)**	Participant	0.33
**State SA (actor)**	Participant	0.21
**State SC (actor)**	Participant	0.18
**State mindfulness—acceptance (partner)**	Participant	0.43
**State mindfulness—attention (partner)**	Participant	0.28
**State SA (partner)**	Participant	0.19
**State SC (partner)**	Participant	0.22
**Residual**	—	1.31

Note: p values reported exactly to three decimals; p < .001 when smaller. Actor = participant’s own state/trait; Partner = partner’s concurrent state/trait.

Exploratory Analyses

We conducted additional exploratory analyses, which we report below.

Age and Relationship Length. We explored age and relationship length as controls. We did not observe any change to the pattern of significant results in terms of the main findings reported in the manuscript, except that there was no longer evidence for a within-person interaction between self-absorption and self-construal. Age and relationship length did not predict compassion. The results are reported below in Table S5.

Table S5a

Hypothesis 1: Partner Ratings of Compassion

Predictor	Estimate	SE	95% CI lower	95% CI upper	df	t	p
**Intercept**	3.44	1.33	0.82	6.05	909	2.57	0.010
**State mindfulness - acceptance (actor)**	0.19	0.07	0.06	0.32	909	2.83	0.005
**State mindfulness - attention (actor)**	0.11	0.07	-0.03	0.24	909	1.54	0.124
**Trait mindfulness -acceptance (actor)**	0.04	0.16	-0.28	0.36	67	0.27	0.788
**Trait mindfulness -attention (actor)**	0.55	0.18	0.19	0.91	67	3.08	0.003
**State mindfulness - acceptance (partner)**	0.28	0.08	0.13	0.42	909	3.66	<.001
**State mindfulness - attention (partner)**	-0.11	0.07	-0.24	0.02	909	-1.6	0.110
**Trait mindfulness**	-0.12	0.17	-0.46	0.23	67	-0.68	0.498

**- acceptance (partner)**							
**Trait mindfulness – attention (partner)**	0.5	0.18	0.14	0.87	67	2.77	0.007
**Age**	0.01	0.02	-0.03	0.05	67	0.43	0.665
**Relationship length**	-0.01	0.02	-0.06	0.03	67	-0.58	0.562

Table S5b

Hypothesis 1: Self Ratings of Compassion

Predictor	Estimate	SE	95% CI lower	95% CI upper	df	t	p
**Intercept**	4.25	1.00	2.29	6.21	912	4.25	<.001
**State mindfulness — acceptance (actor)**	0.26	0.06	0.14	0.38	912	4.12	<.001
**State mindfulness — attention (actor)**	0.09	0.06	-0.03	0.21	912	1.38	0.168
**Trait mindfulness -acceptance (actor)**	0.27	0.13	0.01	0.54	71	2.03	0.046
**Trait mindfulness -attention (actor)**	0.54	0.14	0.26	0.83	71	3.81	<.001
**State mindfulness — acceptance (partner)**	-0.01	0.07	-0.15	0.12	912	-0.18	0.855
**State mindfulness**	-0.02	0.06	-0.14	0.10	912	-0.35	0.727

**— attention (partner)**							
**Trait mindfulness - acceptance (partner)**	-0.32	0.13	-0.58	-0.06	71	-2.49	0.015
**Trait mindfulness – attention (partner)**	0.35	0.14	0.08	0.63	71	2.56	0.013
**Age**	0.004	0.02	-0.03	0.03	71	0.25	0.804
**Relationship length**	0.01	0.02	-0.02	0.05	71	0.67	0.504

Table S5c

Hypothesis 2: Partner Ratings of Compassion

**Outcome**	**Predictor**	**Estimate**	**SE**	**z**	**p**
**Compassion**	Intercept	6.92	1.30	5.37	<.001
**Self-absorption (actor)**	Intercept	9.64	0.52	18.66	<.001
**Self-absorption (partner)**	Intercept	8.12	0.58	14.12	<.001
**Compassion**	Self-absorption (actor)	-0.22	0.05	-4.78	<.001
**Compassion**	Self-absorption (partner)	-0.12	0.05	-2.59	0.010
**Compassion**	State mindfulness — acceptance (actor)	0.06	0.06	1.01	0.313
**Compassion**	State mindfulness — attention (actor)	0.05	0.07	0.72	0.471
**Compassion**	State mindfulness — acceptance (partner)	0.23	0.08	3.05	0.002
**Compassion**	State mindfulness — attention (partner)	-0.15	0.07	-2.31	0.021
**Compassion**	Trait mindfulness — acceptance (actor)	-0.01	0.17	-0.07	0.947
**Compassion**	Trait mindfulness — attention (actor)	0.26	0.20	1.31	0.190
**Compassion**	Trait mindfulness — acceptance (partner)	-0.05	0.18	-0.28	0.781
**Compassion**	Trait mindfulness — attention (partner)	0.37	0.20	1.89	0.060
**Compassion**	Age	0.004	0.02	0.17	0.862
**Compassion**	Relationship length	-0.01	0.02	-0.36	0.719
**Self-absorption (actor)**	State mindfulness — acceptance (actor)	-0.23	0.04	-5.75	<.001

**Self-absorption (actor)**	State mindfulness — attention (actor)	-0.31	0.04	-8.13	<.001
**Self-absorption (actor)**	State mindfulness — acceptance (partner)	-0.07	0.03	-2.53	0.012
**Self-absorption (actor)**	State mindfulness — attention (partner)	-0.02	0.03	-0.67	0.505
**Self-absorption (actor)**	Trait mindfulness — acceptance (actor)	-0.22	0.07	-3.15	0.002
**Self-absorption (actor)**	Trait mindfulness — attention (actor)	-0.91	0.08	-11.81	<.001
**Self-absorption (actor)**	Trait mindfulness — acceptance (partner)	-0.002	0.07	-0.03	0.979
**Self-absorption (actor)**	Trait mindfulness — attention (partner)	-0.14	0.08	-1.63	0.063
**Self-absorption (actor)**	Self-absorption (partner)	0.16	0.02	7.63	<.001
**Self-absorption (actor)**	Age	0.02	0.01	2.37	0.018
**Self-absorption (actor)**	Relationship length	-0.01	0.01	-1.02	0.310
**Self-absorption (partner)**	State mindfulness — acceptance (actor)	-0.04	0.03	-1.72	0.156
**Self-absorption (partner)**	State mindfulness — attention (actor)	-0.01	0.03	-0.28	0.778
**Self-absorption (partner)**	State mindfulness — acceptance (partner)	-0.24	0.04	-6.10	<.001
**Self-absorption (partner)**	State mindfulness — attention (partner)	-0.26	0.04	-6.99	<.001
**Self-absorption (partner)**	Trait mindfulness — acceptance (actor)	0.03	0.07	0.35	0.730
**Self-absorption (partner)**	Trait mindfulness — attention (actor)	0.02	0.08	0.23	0.820
**Self-absorption (partner)**	Trait mindfulness — acceptance (partner)	-0.18	0.07	-2.49	0.013
**Self-absorption (partner)**	Trait mindfulness — attention (partner)	-0.93	0.08	-11.47	<.001
**Self-absorption (partner)**	Age	0.02	0.01	2.12	0.034
**Self-absorption (partner)**	Relationship length	-0.01	0.01	-1.03	0.305

Table S5d

Hypothesis 2: Self Ratings of Compassion

**Outcome**	**Predictor**	**Estimate**	**SE**	**z**	**p**
**Compassion**	Intercept	6.50	1.03	6.30	<.001
**Self-absorption (actor)**	Intercept	9.61	0.51	18.94	<.001
**Self-absorption (partner)**	Intercept	8.08	0.57	14.30	<.001
**Compassion**	Self-absorption (actor)	-0.22	0.04	-5.44	<.001

**Compassion**	Self-absorption (partner)	-0.01	0.04	-0.30	0.762
**Compassion**	State mindfulness — acceptance (actor)	0.19	0.06	3.12	0.002
**Compassion**	State mindfulness — attention (actor)	0.01	0.06	0.10	0.924
**Compassion**	State mindfulness — acceptance (partner)	-0.04	0.07	-0.61	0.540
**Compassion**	State mindfulness — attention (partner)	-0.03	0.06	-0.51	0.613
**Compassion**	Trait mindfulness — acceptance (actor)	0.26	0.14	1.84	0.070
**Compassion**	Trait mindfulness — attention (actor)	0.33	0.15	2.17	0.030
**Compassion**	Trait mindfulness — acceptance (partner)	-0.29	0.14	-2.10	0.035
**Compassion**	Trait mindfulness — attention (partner)	0.31	0.15	2.14	0.032
**Compassion**	Age	0.001	0.02	0.05	0.963
**Compassion**	Relationship length	0.02	0.02	1.03	0.303
**Self-absorption (actor)**	State mindfulness — acceptance (actor)	-0.23	0.04	-5.78	<.001
**Self-absorption (actor)**	State mindfulness — attention (actor)	-0.31	0.04	-8.18	<.001
**Self-absorption (actor)**	State mindfulness — acceptance (partner)	-0.07	0.03	-2.49	0.013
**Self-absorption (actor)**	State mindfulness — attention (partner)	-0.019	0.03	-0.66	0.509
**Self-absorption (actor)**	Trait mindfulness — acceptance (actor)	-0.21	0.07	-3.13	0.002
**Self-absorption (actor)**	Trait mindfulness — attention (actor)	-0.92	0.08	-11.79	<.001
**Self-absorption (actor)**	Trait mindfulness — acceptance (partner)	0.00	0.07	-0.001	0.999
**Self-absorption (actor)**	Trait mindfulness — attention (partner)	-0.14	0.08	-1.82	0.068
**Self-absorption (actor)**	Self-absorption (partner)	0.16	0.02	7.73	<.001
**Self-absorption (actor)**	Age	0.02	0.01	2.40	0.017
**Self-absorption (actor)**	Relationship length	-0.01	0.01	-1.03	0.305
**Self-absorption (partner)**	State mindfulness — acceptance (actor)	-0.04	0.03	-1.39	0.166
**Self-absorption (partner)**	State mindfulness — attention (actor)	-0.01	0.03	-0.25	0.804
**Self-absorption (partner)**	State mindfulness — acceptance (partner)	-0.24	0.04	-6.08	<.001
**Self-absorption (partner)**	State mindfulness — attention (partner)	-0.26	0.04	-7.02	<.001

**Self-absorption (partner)**	Trait mindfulness — acceptance (actor)	0.03	0.07	0.34	0.736
**Self-absorption (partner)**	Trait mindfulness — attention (actor)	0.03	0.08	0.29	0.770
**Self-absorption (partner)**	Trait mindfulness — acceptance (partner)	-0.18	0.07	-2.48	0.013
**Self-absorption (partner)**	Trait mindfulness — attention (partner)	-0.93	0.08	-11.50	<.001
**Self-absorption (partner)**	Age	0.02	0.01	2.13	0.034
**Self-absorption (partner)**	Relationship length	-0.01	0.01	-1.01	0.312

Table S5e

Hypothesis 3: Partner Ratings of Compassion

**Predictor**	**Estimate**	**SE**	**95% CI lower**	**95% CI upper**	**df**	**t**	**p**
**Intercept**	2.00	3.39	-4.66	8.67	903	0.59	0.555
**State mindfulness — acceptance (actor)**	0.08	0.06	-0.05	0.21	903	1.25	0.210
**State mindfulness — attention (actor)**	0.08	0.06	-0.04	0.2	903	1.29	0.198
**Trait mindfulness - acceptance (actor)**	0.04	0.16	-0.27	0.35	61	0.25	0.804
**Trait mindfulness - attention (actor)**	0.21	0.21	-0.22	0.63	61	0.96	0.341
**State mindfulness — acceptance (partner)**	0.22	0.07	0.07	0.37	903	2.95	0.003
**State mindfulness — attention (partner)**	-0.14	0.06	-0.26	-0.01	903	-2.19	0.029
**Trait mindfulness - acceptance (partner)**	-0.21	0.16	-0.54	0.12	61	-1.28	0.206

**Trait mindfulness – attention (partner)**	-0.04	0.22	-0.48	0.41	61	-0.17	0.865
**State self-absorption (actor)**	-0.15	0.06	-0.26	-0.04	903	-2.65	0.008
**State self-construal (actor)**	0.1	0.05	0.01	0.19	903	2.15	0.031
**Trait self-absorption (actor)**	0.16	0.49	-0.83	1.14	61	0.32	0.753
**Trait self-construal (actor)**	0.28	0.21	-0.13	0.69	61	1.37	0.177
**State self-absorption (partner)**	-0.07	0.05	-0.16	0.02	903	-1.56	0.120
**State self-construal (partner)**	0.14	0.06	0.03	0.26	903	2.46	0.014
**Trait self-absorption (partner)**	0.52	0.47	-0.42	1.46	61	1.10	0.276
**Trait self-construal (partner)**	0.62	0.2	0.22	1.02	61	3.13	0.003
**Age**	0.01	0.02	-0.02	0.05	61	0.74	0.465
**Relationship length**	-0.01	0.02	-0.05	0.03	61	-0.59	0.556
**State SA × State SC (actor)**	-0.02	0.02	-0.06	0.02	903	-1.10	0.272
**Trait SA × Trait SC (actor)**	-0.03	0.05	-0.14	0.08	61	-0.55	0.584
**State SA × State SC (partner)**	-0.01	0.02	-0.05	0.03	903	-0.3	0.761
**Trait SA × Trait SC (partner)**	-0.11	0.05	-0.21	-0.01	61	-2.16	0.034

Table S5f

Hypothesis 3: Self Ratings of Compassion

Predictor	Estimate	SE	95% CI lower	95% CI upper	df	t	p
**Intercept**	5.83	2.58	0.76	10.89	906	2.26	0.024
**State mindfulness —**	0.11	0.06	-0.01	0.23	906	1.84	0.067

**acceptance (actor)**							
**State mindfulness — attention (actor)**	0.02	0.06	-0.09	0.14	906	0.36	0.716
**Trait mindfulness - acceptance (actor)**	0.3	0.12	0.06	0.55	65	2.46	0.017
**Trait mindfulness - attention (actor)**	0.27	0.16	-0.05	0.58	65	1.66	0.101
**State mindfulness — acceptance (partner)**	0	0.06	-0.12	0.12	906	0.01	0.991
**State mindfulness — attention (partner)**	-0.01	0.05	-0.11	0.09	906	-0.21	0.836
**Trait mindfulness - acceptance (partner)**	-0.26	0.12	-0.49	-0.02	65	-2.17	0.033
**Trait mindfulness – attention (partner)**	0.09	0.16	-0.24	0.41	65	0.53	0.597
**State self-absorption (actor)**	-0.14	0.04	-0.23	-0.06	906	-3.27	0.001
**State self-construal (actor)**	0.33	0.04	0.25	0.4	906	8.67	<.001
**Trait self-absorption (actor)**	-0.77	0.37	-1.5	-0.03	65	-2.09	0.040
**Trait self-construal (actor)**	0.04	0.16	-0.27	0.35	65	0.28	0.780
**State self-absorption (partner)**	0.06	0.04	-0.03	0.14	906	1.35	0.177
**State self-construal (partner)**	0.02	0.05	-0.07	0.11	906	0.53	0.595

**Trait self-absorption (partner)**	0.06	0.33	-0.6	0.72	65	0.17	0.862
**Trait self-construal (partner)**	0.13	0.14	-0.16	0.41	65	0.9	0.371
**Age**	0	0.01	-0.03	0.03	65	-0.21	0.836
**Relationship length**	0.02	0.02	-0.01	0.05	65	1.12	0.269
**State SA × State SC (actor)**	0.02	0.02	-0.02	0.05	906	0.91	0.361
**Trait SA × Trait SC (actor)**	0.08	0.04	0	0.16	65	1.9	0.062
**State SA × State SC (partner)**	-0.04	0.02	-0.08	-0.01	906	-2.49	0.013
**Trait SA × Trait SC (partner)**	-0.02	0.04	-0.09	0.06	65	-0.44	0.664

Self-construal as Moderator. The results of our moderation test prompted an exploratory investigation of the role of interdependent self-construal. In line with past research, we tested the possibility that self-construal moderated the relationship between mindfulness and compassion without self-absorption as a mediator, but did not find support for this. The results are reported below in Table S6.

Table S6a

Partner Ratings of Compassion

**Predictor**	**Estimate**	**SE**	**95% CI lower**	**95% CI upper**	**df**	**t**	**p**
**Intercept**	11.75	5.86	0.26	23.24	1042	2.01	0.045
**State mindfulness — acceptance (actor)**	0.12	0.06	0	0.24	1042	1.98	0.048
**State mindfulness — attention (actor)**	0.16	0.05	0.07	0.25	1042	3.51	<.001
**Trait mindfulness - acceptance (actor)**	0.07	0.06	-0.05	0.19	1042	1.08	0.281
**Trait mindfulness - attention (actor)**	-0.26	0.58	-1.41	0.89	82	-0.45	0.656

**State mindfulness — acceptance (partner)**	-0.50	0.42	-1.34	0.33	82	-1.2	0.234
**State mindfulness — attention (partner)**	-0.72	0.68	-2.08	0.64	82	-1.05	0.298
**Trait mindfulness - acceptance (partner)**	0.25	0.07	0.11	0.38	1042	3.66	<.001
**Trait mindfulness – attention (partner)**	0.20	0.05	0.1	0.31	1042	3.77	<.001
**State self-construal (actor)**	-0.10	0.06	-0.22	0.01	1042	-1.75	0.080
**Trait self-construal (actor)**	0.98	0.61	-0.24	2.19	82	1.6	0.114
**State self-construal (partner)**	-0.23	0.41	-1.06	0.59	82	-0.57	0.572
**Trait self-construal (partner)**	-1.48	0.66	-2.79	-0.16	82	-2.23	0.029
**State acc × state SC (actor)**	0.02	0.03	-0.04	0.07	1042	0.6	0.549
**State att × state SC (actor)**	0.01	0.03	-0.05	0.08	1042	0.4	0.690
**Trait acc × trait SC (actor)**	0.02	0.06	-0.11	0.15	82	0.27	0.786
**Trait att × trait SC (actor)**	0.13	0.08	-0.03	0.29	82	1.58	0.118
**State acc × state SC (partner)**	0.02	0.03	-0.04	0.07	1042	0.57	0.572
**State att × state SC (partner)**	-0.01	0.03	-0.08	0.05	1042	-0.36	0.722
**Trait acc × trait SC (partner)**	-0.14	0.07	-0.27	0	82	-2	0.049
**Trait att × trait SC (partner)**	0.22	0.08	0.07	0.38	82	2.89	0.005

Table S6b

Self Ratings of Compassion

Predictor	Estimate	SE	95% CI lower	95% CI upper	df	t	p
**Intercept**	-0.9	4.23	-9.19	7.4	1037	-0.21	0.832
**State mindfulness — acceptance (actor)**	0.16	0.06	0.05	0.27	1037	2.85	0.004
**State mindfulness — attention (actor)**	0.34	0.04	0.26	0.41	1037	8.87	<.001
**Trait mindfulness - acceptance (actor)**	0.07	0.05	-0.04	0.17	1037	1.22	0.224
**Trait mindfulness - attention (actor)**	0.9	0.47	-0.03	1.83	85	1.92	0.058
**State mindfulness — acceptance (partner)**	0.67	0.31	0.05	1.28	85	2.16	0.034
**State mindfulness — attention (partner)**	0.35	0.51	-0.67	1.37	85	0.68	0.498
**Trait mindfulness - acceptance (partner)**	-0.04	0.06	-0.15	0.08	1037	-0.66	0.511
**Trait mindfulness – attention (partner)**	0.03	0.04	-0.05	0.12	1037	0.73	0.467
**State self-construal (actor)**	-0.03	0.05	-0.13	0.08	1037	-0.51	0.611
**Trait self-construal (actor)**	0.24	0.44	-0.64	1.12	85	0.54	0.588
**State self-construal (partner)**	0.09	0.3	-0.5	0.69	85	0.32	0.750
**Trait self-construal (partner)**	-0.24	0.48	-1.19	0.72	85	-0.5	0.622
**State acc × state SC (actor)**	-0.03	0.02	-0.07	0.02	1037	-1.07	0.283

**State att × state SC (actor)**	0.02	0.03	-0.04	0.07	1037	0.58	0.564
**Trait acc × trait SC (actor)**	-0.08	0.05	-0.18	0.02	85	-1.5	0.137
**Trait att × trait SC (actor)**	0.01	0.06	-0.11	0.13	85	0.12	0.904
**State acc × state SC (partner)**	0.04	0.03	-0.01	0.09	1037	1.57	0.117
**State att × state SC (partner)**	-0.03	0.03	-0.09	0.03	1037	-0.96	0.338
**Trait acc × trait SC (partner)**	-0.05	0.05	-0.15	0.04	85	-1.11	0.270
**Trait att × trait SC (partner)**	0.05	0.06	-0.06	0.16	85	0.85	0.399

Self-construal as Mediator. We further tested the possibility that self-construal mediated the link between mindfulness and compassion. We used the same statistical procedure as for the mediation of self-absorption which is described in the main text. We found some support for this in terms of self-ratings of compassion, in which there was an indirect effect from within-person acceptance (ab=0.06, 95% BCa CI[0.04, 0.08]) and between-person attention (ab=0.10, 95% BCa CI[0.01, 0.20]) through self-construal to self-rated compassion. The results of the models are reported below in Table S7. The indirect effects are further described in Figures S3 and S4.

Table S7a

Partner Ratings of Compassion

Outcome	Predictor	Estimate	SE	95% CI lower	95% CI upper	df	t	p
**Compassion**	Intercept	2.42	1.26	0.08	4.67	6096	1.91	0.056
**Self-construal (actor)**	Intercept	5.07	1.01	3.19	6.84	6096	5.02	<.001
**Self-construal (partner)**	Intercept	4.71	1.03	3	6.4	6096	4.57	<.001
**Compassion**	Self-construal (actor)	0.08	0.03	-0.01	0.16	6096	2.29	0.022
**Compassion**	Self-construal (partner)	0.12	0.04	0.04	0.22	6096	2.69	0.007
**Compassion**	State mindfulness — acceptance (actor)	0.14	0.06	0.01	0.28	6096	2.43	0.015

**Compassion**	State mindfulness — attention (actor)	0.07	0.06	-0.06	0.2	6096	1.14	0.256
**Compassion**	State mindfulness — acceptance (partner)	0.24	0.07	0.11	0.42	6096	3.44	0.001
**Compassion**	State mindfulness — attention (partner)	-0.1	0.06	-0.22	0.02	6096	-1.71	0.087
**Compassion**	Trait mindfulness — acceptance (actor)	0.03	0.17	-0.3	0.39	6096	0.18	0.855
**Compassion**	Trait mindfulness — attention (actor)	0.53	0.18	0.11	0.84	6096	2.87	0.004
**Compassion**	Trait mindfulness — acceptance (partner)	-0.04	0.18	-0.36	0.37	6096	-0.21	0.831
**Compassion**	Trait mindfulness — attention (partner)	0.32	0.19	-0.02	0.74	6096	1.71	0.088
**Self-construal (actor)**	State mindfulness — acceptance (actor)	0.21	0.04	0.14	0.29	6096	5.96	<.001
**Self-construal (actor)**	State mindfulness — attention (actor)	0.06	0.04	-0.04	0.14	6096	1.48	0.138
**Self- construal (actor)**	State mindfulness — acceptance (partner)	0.03	0.03	-0.03	0.09	6096	0.96	0.339
**Self-construal (actor)**	State mindfulness — attention (partner)	0.02	0.04	-0.06	0.1	6096	0.39	0.697
**Self-construal (actor)**	Trait mindfulness — acceptance (actor)	0.23	0.13	-0.07	0.56	6096	1.72	0.085
**Self-construal (actor)**	Trait mindfulness — attention (actor)	0.35	0.14	0.01	0.66	6096	2.42	0.015
**Self-construal (actor)**	Trait mindfulness — acceptance (partner)	-0.18	0.13	-0.51	0.1	6096	-1.41	0.158

**Self-construal (actor)**	Trait mindfulness — attention (partner)	0.39	0.14	0.07	0.7	6096	2.71	0.007
**Self-construal (actor)**	Self-construal (partner)	0.08	0.02	0	0.17	6096	4.03	<.001
**Self-construal (partner)**	State mindfulness — acceptance (actor)	0.04	0.03	-0.02	0.1	6096	1.27	0.204
**Self-construal (partner)**	State mindfulness — attention (actor)	0	0.04	-0.07	0.08	6096	-0.05	0.962
**Self-construal (partner)**	State mindfulness — acceptance (partner)	0.23	0.04	0.16	0.31	6096	6.22	<.001
**Self-construal (partner)**	State mindfulness — attention (partner)	0.06	0.04	-0.03	0.14	6096	1.52	0.129
**Self-construal (partner)**	Trait mindfulness — acceptance (actor)	-0.25	0.13	-0.59	0.01	6096	-1.9	0.057
**Self-construal (partner)**	Trait mindfulness — attention (actor)	0.38	0.15	0.07	0.69	6096	2.6	0.009
**Self-construal (partner)**	Trait mindfulness — acceptance (partner)	0.26	0.13	-0.03	0.59	6096	1.95	0.051
**Self-construal (partner)**	Trait mindfulness — attention (partner)	0.32	0.15	-0.01	0.61	6096	2.18	0.029

Figure S3

Indirect Effects of Actor Mindfulness on Partner Ratings of Compassion via Self–Construal, Controlling for Partner Effects

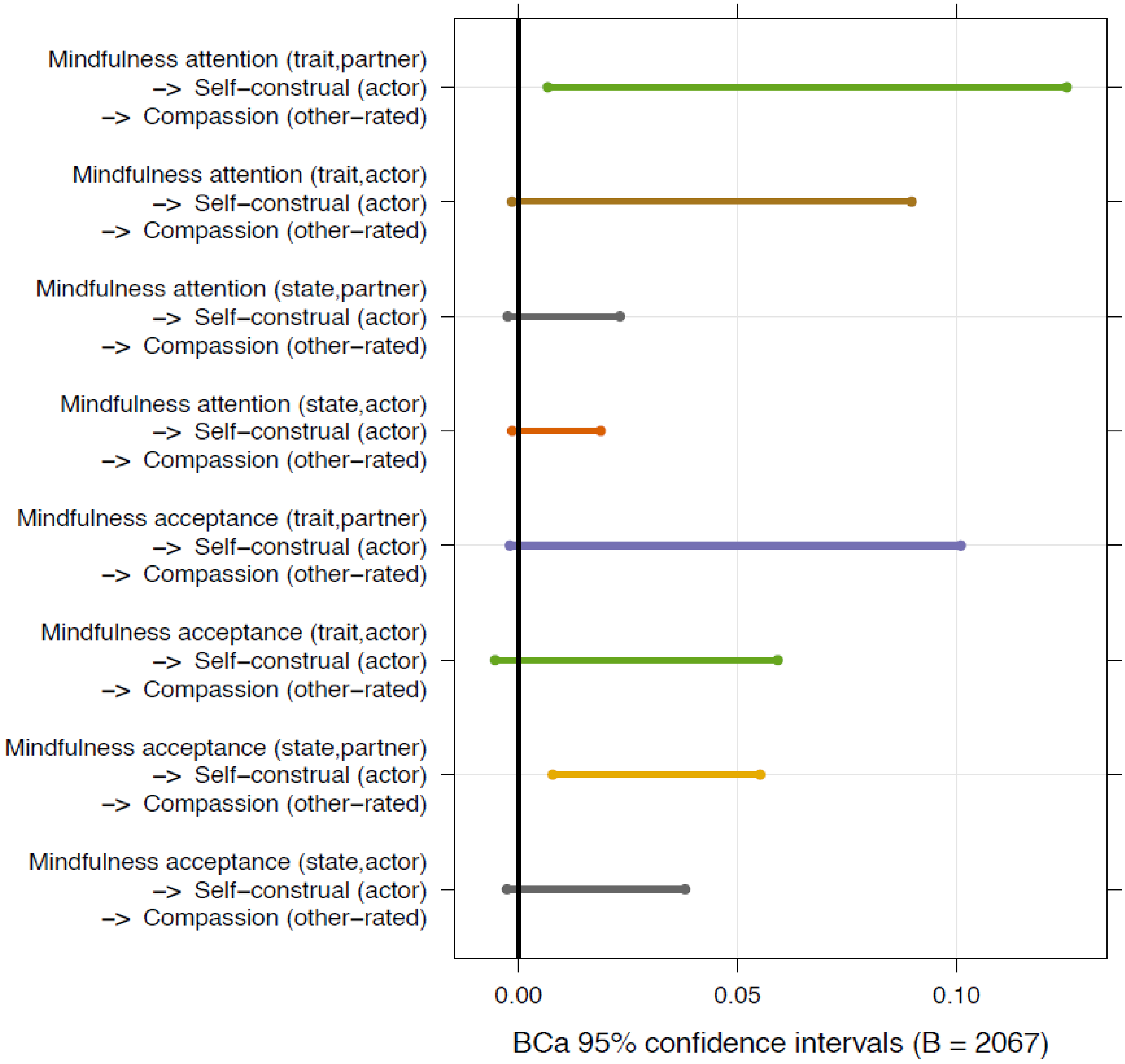

Note. N=226 participants

Table S7b

Self Ratings of Compassion

Outcome	Predictor	Estimate	SE	95% CI lower	95% CI upper	df	t	p
**Compassion**	Intercept	3.29	0.97	1.56	4.93	6094	3.41	<.001

**Self-construal (actor)**	Intercept	5.13	0.96	3.16	6.94	6094	5.34	<.001
**Self-construal (partner)**	Intercept	4.73	0.98	2.99	6.59	6094	4.84	<.001
**Compassion**	Self-construal (actor)	0.27	0.03	0.2	0.34	6094	9.56	<.001
**Compassion**	Self-construal (partner)	-0.07	0.03	-0.16	0.01	6094	-2.09	0.037
**Compassion**	State mindfulness — acceptance (actor)	0.2	0.06	0.08	0.33	6094	3.39	<.001
**Compassion**	State mindfulness — attention (actor)	0.05	0.05	-0.06	0.15	6094	0.94	0.349
**Compassion**	State mindfulness — acceptance (partner)	-0.01	0.06	-0.13	0.11	6094	-0.2	0.844
**Compassion**	State mindfulness — attention (partner)	-0.02	0.05	-0.13	0.09	6094	-0.36	0.715
**Compassion**	Trait mindfulness — acceptance (actor)	0.19	0.13	-0.03	0.44	6094	1.46	0.146
**Compassion**	Trait mindfulness — attention (actor)	0.49	0.14	0.28	0.71	6094	3.56	<.001
**Compassion**	Trait mindfulness — acceptance (partner)	-0.16	0.13	-0.38	0.06	6094	-1.29	0.199
**Compassion**	Trait mindfulness — attention (partner)	0.21	0.14	-0.03	0.45	6094	1.52	0.128
**Self-construal (actor)**	State mindfulness — acceptance (actor)	0.21	0.04	0.14	0.29	6094	5.93	<.001
**Self-construal (actor)**	State mindfulness — attention (actor)	0.06	0.04	-0.04	0.15	6094	1.62	0.106
**Self- construal (actor)**	State mindfulness — acceptance (partner)	0.03	0.03	-0.03	0.09	6094	0.89	0.375
**Self-construal (actor)**	State mindfulness —	0.02	0.04	-0.06	0.1	6094	0.51	0.607

	attention (partner)							
**Self-construal (actor)**	Trait mindfulness — acceptance (actor)	0.22	0.13	-0.07	0.55	6094	1.72	0.085
**Self-construal (actor)**	Trait mindfulness — attention (actor)	0.34	0.14	0.01	0.66	6094	2.4	0.016
**Self-construal (actor)**	Trait mindfulness — acceptance (partner)	-0.19	0.13	-0.51	0.09	6094	-1.46	0.144
**Self-construal (actor)**	Trait mindfulness — attention (partner)	0.38	0.14	0.07	0.72	6094	2.67	0.008
**Self-construal (actor)**	Self-construal (partner)	0.09	0.02	0	0.18	6094	4.64	<.001
**Self-construal (partner)**	State mindfulness — acceptance (actor)	0.04	0.03	-0.03	0.1	6094	1.18	0.239
**Self-construal (partner)**	State mindfulness — attention (actor)	0	0.04	-0.07	0.07	6094	-0.04	0.967
**Self-construal (partner)**	State mindfulness — acceptance (partner)	0.23	0.04	0.16	0.31	6094	6.19	<.001
**Self-construal (partner)**	State mindfulness — attention (partner)	0.06	0.04	-0.04	0.14	6094	1.52	0.128
**Self-construal (partner)**	Trait mindfulness — acceptance (actor)	-0.26	0.13	-0.58	0	6094	-1.97	0.049
**Self-construal (partner)**	Trait mindfulness — attention (actor)	0.36	0.15	0.05	0.69	6094	2.45	0.015
**Self-construal (partner)**	Trait mindfulness — acceptance (partner)	0.26	0.13	-0.04	0.56	6094	1.99	0.047
**Self-construal (partner)**	Trait mindfulness — attention (partner)	0.32	0.14	-0.01	0.63	6094	2.21	0.027

Figure S4

Indirect Effects of Actor Mindfulness on Self Ratings of Compassion via Self–Construal, Controlling for Partner Effects

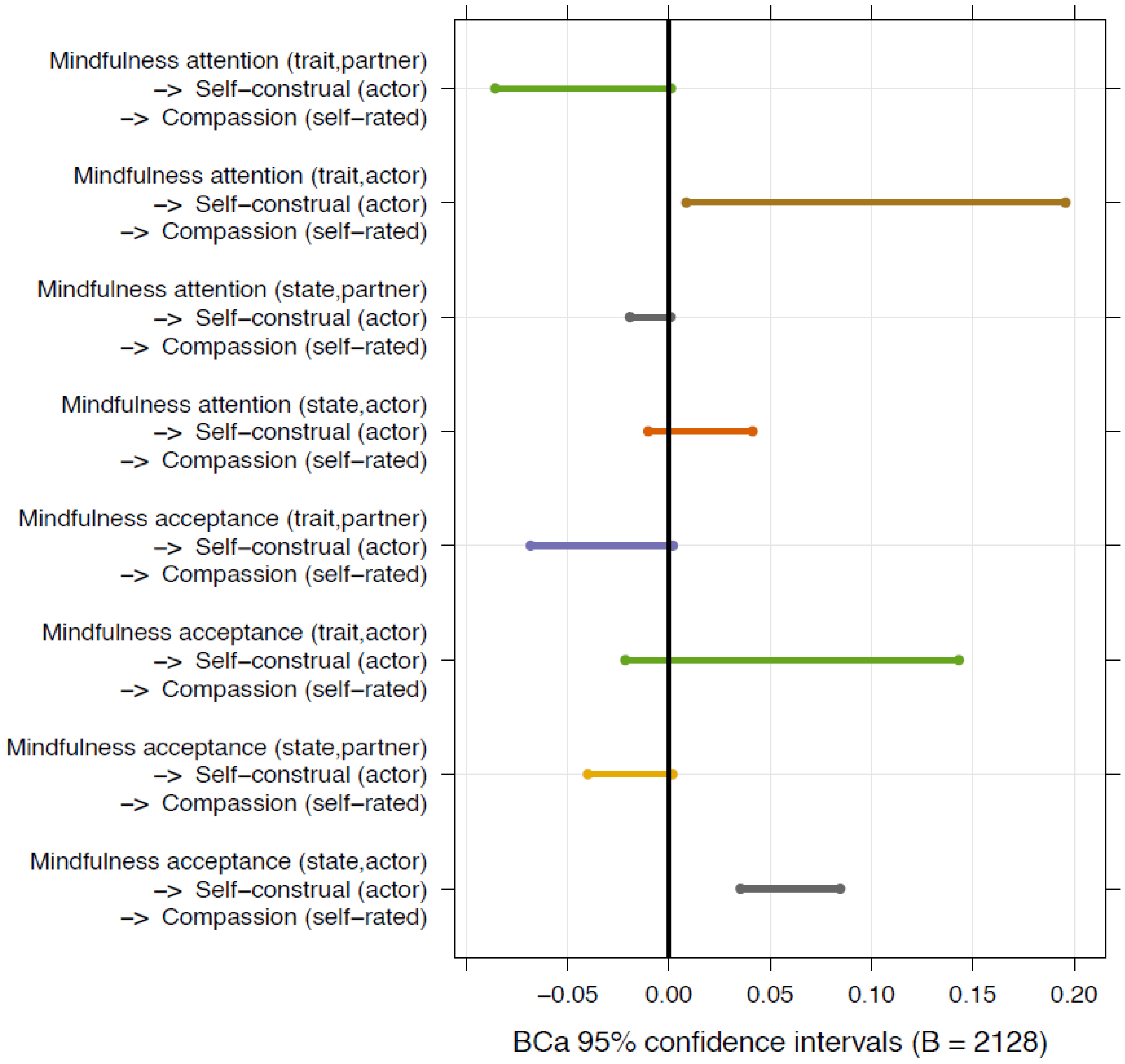

Note. N=226 participants

Mindfulness and Reporting Unpleasant Feelings. As noted in the main text, we analyzed compassion only on days when an opportunity for compassion occurred, and therefore dropped from the analysis any N/A responses to the compassion questions (i.e., "I did not experience any unpleasant feelings today/My partner did not show that they experienced any unpleasant feelings today"). However, to better understand the dynamics at

play, we also investigated how mindfulness related to selecting these responses. Mindfulness at both the within- and between-person levels did generally predict selecting both that one did not experience any unpleasant feelings that day, and that their partner did not show that they experienced any unpleasant feelings that day. The results are reported below in Table S8.

Table S8a

Rating One's Partner as N/A for Compassion Due to Their Not Showing Unpleasant Emotions

Predictor	Estimate	SE	95% CI lower	95% CI upper	df	t	p
**Intercept**	-0.35	0.16	-0.67	-0.04	2321	-2.2	0.028
**State mindfulness - acceptance (actor)**	0.02	0.01	0	0.04	2321	2.48	0.013
**State mindfulness - attention (actor)**	0.04	0.01	0.02	0.06	2321	4.19	<.001
**Trait mindfulness -acceptance (actor)**	0.02	0.02	-0.02	0.06	110	0.97	0.333
**Trait mindfulness -attention (actor)**	0.05	0.02	0	0.1	110	2.12	0.036
**State mindfulness - acceptance (partner)**	0.04	0.01	0.02	0.06	2321	4.76	<.001
**State mindfulness - attention (partner)**	0.02	0.01	0.01	0.04	2321	2.77	0.006
**Trait mindfulness**	0.08	0.02	0.04	0.13	110	3.77	<.001

**- acceptance (partner)**							
**Trait mindfulness – attention (partner)**	0.04	0.02	-0.01	0.09	110	1.58	0.116

Note. N/A=1, not-N/A=0

Table S8b

Rating Oneself as N/A for Compassion Due to Not Experiencing Unpleasant Emotions

**Predictor**	**Estimate**	**SE**	**95% CI lower**	**95% CI upper**	**df**	**t**	**p**
**Intercept**	-0.43	0.16	-0.74	-0.11	2321	-2.68	0.007
**State mindfulness - acceptance (actor)**	0.08	0.01	0.06	0.1	2321	7.99	<.001
**State mindfulness - attention (actor)**	0.03	0.01	0.02	0.05	2321	4.32	<.001
**Trait mindfulness -acceptance (actor)**	0.08	0.02	0.04	0.13	110	3.64	<.001
**Trait mindfulness -attention (actor)**	0.07	0.02	0.02	0.12	110	2.73	0.007
**State mindfulness - acceptance (partner)**	0.02	0.01	0	0.03	2321	2.33	0.020
**State mindfulness - attention (partner)**	0.01	0.01	-0.01	0.02	2321	0.88	0.379

**Trait mindfulness - acceptance (partner)**	0.06	0.02	0.02	0.11	110	2.88	0.005
**Trait mindfulness – attention (partner)**	-0.01	0.02	-0.05	0.04	110	-0.2	0.839

Note. N/A=1, not-N/A=0

Facets of Compassion. Since the compassion measure included items for both noticing ("During time spent with my partner today, they (I) noticed and were (was) sensitive to any unpleasant feelings that I (they) experienced") and acting ("During time spent with my partner today, my partner (I) treated me (my partner) with feelings of support, helpfulness and encouragement"), we examined item-level associations between mindfulness and compassion. When compassion was partner-rated, only the action item was predicted by mindfulness. However, when compassion was self-rated, both items were predicted by mindfulness. Further, both items were predicted by self-absorption. The results are reported below in Table S9.

Table S9a

Hypothesis 1: Partner Ratings of Compassion- Action Item

Predictor	Estimate	SE	95% CI lower	95% CI upper	df	t	p
**Intercept**	1.97	0.59	0.81	3.12	1048	3.33	<.001
**State mindfulness - acceptance (actor)**	0.14	0.03	0.08	0.2	1048	4.34	<.001
**State mindfulness - attention (actor)**	0.02	0.03	-0.05	0.08	1048	0.51	0.608
**Trait mindfulness**	-0.08	0.08	-0.23	0.07	88	-1.06	0.293

**-acceptance (actor)**							
**Trait mindfulness -attention (actor)**	0.39	0.08	0.23	0.56	88	4.79	<.001
**State mindfulness - acceptance (partner)**	0.2	0.03	0.13	0.27	1048	5.82	<.001
**State mindfulness - attention (partner)**	0.01	0.03	-0.05	0.07	1048	0.32	0.746
**Trait mindfulness - acceptance (partner)**	-0.08	0.08	-0.24	0.08	88	-0.95	0.345
**Trait mindfulness – attention (partner)**	0.28	0.08	0.12	0.45	88	3.41	<.001

Table S9b

Hypothesis 1: Partner Ratings of Compassion- Noticing Item

Predictor	Estimate	SE	95% CI lower	95% CI upper	df	t	p
**Intercept**	2.33	0.65	1.05	3.61	1048	3.58	<.001
**State mindfulness - acceptance (actor)**	0.05	0.04	-0.03	0.12	1048	1.26	0.206
**State mindfulness - attention (actor)**	0.03	0.04	-0.04	0.11	1048	0.87	0.386

**Trait mindfulness -acceptance (actor)**	0.02	0.09	-0.15	0.2	88	0.28	0.783
**Trait mindfulness -attention (actor)**	0.13	0.1	-0.07	0.32	88	1.32	0.190
**State mindfulness - acceptance (partner)**	0.1	0.04	0.02	0.19	1048	2.45	0.015
**State mindfulness - attention (partner)**	-0.1	0.04	-0.18	-0.02	1048	-2.52	0.012
**Trait mindfulness - acceptance (partner)**	-0.05	0.1	-0.24	0.14	88	-0.53	0.600
**Trait mindfulness – attention (partner)**	0.18	0.1	-0.02	0.38	88	1.79	0.078

Table S9c

Hypothesis 1: Self Ratings of Compassion- Action Item

**Predictor**	**Estimate**	**SE**	**95% CI lower**	**95% CI upper**	**df**	**t**	**p**
**Intercept**	2.01	0.54	0.95	3.07	1048	3.72	<.001
**State mindfulness - acceptance (actor)**	0.15	0.03	0.09	0.2	1048	4.78	<.001
**State mindfulness**	0.07	0.03	0.01	0.14	1048	2.15	0.032

**- attention (actor)**							
**Trait mindfulness -acceptance (actor)**	0.13	0.07	-0.01	0.27	88	1.79	0.078
**Trait mindfulness -attention (actor)**	0.31	0.08	0.15	0.46	88	3.88	<.001
**State mindfulness - acceptance (partner)**	0.04	0.03	-0.01	0.1	1048	1.48	0.139
**State mindfulness - attention (partner)**	0	0.03	-0.07	0.07	1048	-0.04	0.969
**Trait mindfulness - acceptance (partner)**	-0.13	0.08	-0.29	0.02	88	-1.75	0.083
**Trait mindfulness – attention (partner)**	0.23	0.08	0.07	0.38	88	2.83	0.006

Table S9d

Hypothesis 1: Self Ratings of Compassion- Noticing Item

**Predictor**	**Estimate**	**SE**	**95% CI lower**	**95% CI upper**	**df**	**t**	**p**
**Intercept**	2.57	0.54	1.51	3.63	1043	4.76	<.001
**State mindfulness - acceptance (actor)**	0.11	0.04	0.03	0.18	1043	2.83	0.005

**State mindfulness - attention (actor)**	-0.01	0.04	-0.08	0.07	1043	-0.14	0.887
**Trait mindfulness -acceptance (actor)**	0.16	0.08	0.01	0.32	91	2.1	0.039
**Trait mindfulness -attention (actor)**	0.18	0.08	0.01	0.34	91	2.11	0.037
**State mindfulness - acceptance (partner)**	-0.05	0.04	-0.13	0.03	1043	-1.32	0.186
**State mindfulness - attention (partner)**	-0.04	0.04	-0.12	0.03	1043	-1.17	0.241
**Trait mindfulness - acceptance (partner)**	-0.19	0.08	-0.34	-0.04	91	-2.52	0.013
**Trait mindfulness – attention (partner)**	0.14	0.08	-0.02	0.3	91	1.75	0.084

Table S9e

Hypothesis 2: Partner Ratings of Compassion- Action Item

Predictor	Estimate	SE	95% CI lower	95% CI upper	df	t	p
**Intercept**	4.86	1.19	2.54	7.19	1046	4.11	<.001
**State mindfulness -**	0.08	0.03	0.02	0.14	1046	2.52	0.012

**acceptance (actor)**							
**State mindfulness - attention (actor)**	0.00	0.03	-0.06	0.07	1046	0.13	0.900
**Trait mindfulness -acceptance (actor)**	-0.10	0.08	-0.24	0.05	86	-1.27	0.207
**Trait mindfulness -attention (actor)**	0.31	0.11	0.1	0.52	86	2.9	0.005
**State mindfulness - acceptance (partner)**	0.19	0.04	0.12	0.26	1046	5.27	<.001
**State mindfulness - attention (partner)**	-0.01	0.03	-0.07	0.05	1046	-0.45	0.652
**Trait mindfulness - acceptance (partner)**	-0.09	0.08	-0.25	0.07	86	-1.12	0.266
**Trait mindfulness – attention (partner)**	0.04	0.11	-0.18	0.25	86	0.34	0.732
**State self-absorption (actor)**	-0.08	0.02	-0.12	-0.03	1046	-3.2	0.001
**Trait self-absorption (actor)**	-0.05	0.07	-0.19	0.1	86	-0.63	0.53
**State self-absorption (partner)**	-0.08	0.03	-0.13	-0.03	1046	-3.01	0.003
**Trait self-absorption (partner)**	-0.26	0.07	-0.41	-0.12	86	-3.62	<.001

Table S9f

Hypothesis 2: Partner Ratings of Compassion- Noticing Item

Predictor	Estimate	SE	95% CI lower	95% CI upper	df	t	p
**Intercept**	5.4	1.33	2.79	8	1046	4.06	<.001
**State mindfulness - acceptance (actor)**	0.01	0.04	-0.08	0.09	1046	0.13	0.896
**State mindfulness - attention (actor)**	0.02	0.04	-0.06	0.09	1046	0.42	0.677
**Trait mindfulness -acceptance (actor)**	-0.01	0.09	-0.19	0.16	86	-0.14	0.890
**Trait mindfulness -attention (actor)**	-0.08	0.12	-0.33	0.17	86	-0.65	0.519
**State mindfulness - acceptance (partner)**	0.09	0.04	0.01	0.17	1046	2.21	0.027
**State mindfulness - attention (partner)**	-0.09	0.04	-0.17	-0.01	1046	-2.33	0.020
**Trait mindfulness - acceptance (partner)**	-0.07	0.09	-0.26	0.12	86	-0.73	0.470
**Trait mindfulness – attention (partner)**	0.05	0.13	-0.2	0.3	86	0.38	0.707

**State self-absorption (actor)**	-0.1	0.03	-0.17	-0.03	1046	-2.95	0.003
**Trait self-absorption (actor)**	-0.21	0.08	-0.37	-0.04	86	-2.42	0.017
**State self-absorption (partner)**	0.02	0.03	-0.04	0.08	1046	0.75	0.456
**Trait self-absorption (partner)**	-0.12	0.08	-0.29	0.04	86	-1.47	0.146

Table S9g

Hypothesis 2: Self Ratings of Compassion- Action Item

Predictor	Estimate	SE	95% CI lower	95% CI upper	df	t	p
**Intercept**	3.47	1.03	1.46	5.48	1046	3.38	<.001
**State mindfulness - acceptance (actor)**	0.10	0.03	0.04	0.16	1046	3.43	<.001
**State mindfulness - attention (actor)**	0.04	0.03	-0.02	0.11	1046	1.31	0.192
**Trait mindfulness -acceptance (actor)**	0.10	0.07	-0.04	0.24	86	1.49	0.141
**Trait mindfulness -attention (actor)**	0.30	0.1	0.11	0.49	86	3.14	0.002
**State mindfulness - acceptance (partner)**	0.04	0.03	-0.01	0.1	1046	1.55	0.121

**State mindfulness - attention (partner)**	-0.01	0.03	-0.07	0.06	1046	-0.15	0.879
**Trait mindfulness - acceptance (partner)**	-0.15	0.08	-0.3	0	86	-1.94	0.055
**Trait mindfulness – attention (partner)**	0.08	0.1	-0.12	0.27	86	0.77	0.444
**State self-absorption (actor)**	-0.11	0.03	-0.16	-0.06	1046	-3.98	<.001
**Trait self-absorption (actor)**	0.03	0.07	-0.1	0.17	86	0.48	0.629
**State self-absorption (partner)**	0.02	0.02	-0.03	0.06	1046	0.71	0.477
**Trait self-absorption (partner)**	-0.18	0.07	-0.32	-0.05	86	-2.73	0.008

Table S9h

Hypothesis 2: Self Ratings of Compassion- Noticing Item

Predictor	Estimate	SE	95% CI lower	95% CI upper	df	t	p
**Intercept**	3.85	1.24	1.42	6.27	1041	3.11	0.002
**State mindfulness - acceptance (actor)**	0.09	0.04	0.01	0.16	1041	2.16	0.031
**State mindfulness - attention (actor)**	-0.02	0.04	-0.1	0.05	1041	-0.62	0.533

**Trait mindfulness -acceptance (actor)**	0.17	0.09	0	0.34	89	1.98	0.051
**Trait mindfulness -attention (actor)**	0.13	0.11	-0.09	0.36	89	1.18	0.242
**State mindfulness - acceptance (partner)**	-0.05	0.04	-0.13	0.02	1041	-1.34	0.180
**State mindfulness - attention (partner)**	-0.03	0.04	-0.11	0.04	1041	-0.87	0.383
**Trait mindfulness - acceptance (partner)**	-0.2	0.08	-0.37	-0.03	89	-2.39	0.019
**Trait mindfulness – attention (partner)**	0.02	0.12	-0.21	0.25	89	0.17	0.864
**State self-absorption (actor)**	-0.07	0.03	-0.12	-0.01	1041	-2.38	0.018
**Trait self-absorption (actor)**	-0.01	0.08	-0.16	0.14	89	-0.16	0.877
**State self-absorption (partner)**	0.03	0.03	-0.02	0.09	1041	1.19	0.234
**Trait self-absorption (partner)**	-0.13	0.08	-0.29	0.03	89	-1.61	0.112

The Effect of Time on Compassion Ratings. Since participants rated both themselves and their partners on the same constructs over time, we tested to see if there was a possible effect of time on compassion ratings. We found an effect of survey day on compassion ratings for both partner- and self-rated compassion, though

including survey day did not alter the pattern of significant results reported in the main text. The results are reported below in Table S10.

Table S10a

Hypothesis 1: Partner Ratings of Compassion

Predictor	Estimate	SE	95% CI lower	95% CI upper	df	t	p
**Intercept**	4.56	1.16	2.29	6.83	1047	3.94	<.001
**State mindfulness - acceptance (actor)**	0.17	0.06	0.05	0.29	1047	2.8	0.005
**State mindfulness - attention (actor)**	0.06	0.06	-0.07	0.19	1047	0.93	0.352
**Trait mindfulness -acceptance (actor)**	-0.06	0.15	-0.36	0.23	88	-0.43	0.669
**Trait mindfulness -attention (actor)**	0.52	0.16	0.19	0.84	88	3.14	0.002
**State mindfulness - acceptance (partner)**	0.29	0.07	0.16	0.43	1047	4.26	<.001
**State mindfulness - attention (partner)**	-0.09	0.06	-0.2	0.03	1047	-1.43	0.153
**Trait mindfulness - acceptance (partner)**	-0.13	0.16	-0.44	0.19	88	-0.78	0.437

**Trait mindfulness – attention (partner)**	0.47	0.17	0.14	0.8	88	2.8	0.006
**Day of survey**	-0.03	0.01	-0.06	-0.01	1047	-2.54	0.011

Table S10b

Hypothesis 1: Self Ratings of Compassion

**Predictor**	**Estimate**	**SE**	**95% CI lower**	**95% CI upper**	**df**	**t**	**p**
**Intercept**	5.61	0.89	3.86	7.36	1042	6.29	<.001
**State mindfulness - acceptance (actor)**	0.24	0.06	0.13	0.35	1042	4.16	<.001
**State mindfulness - attention (actor)**	0.1	0.06	-0.01	0.21	1042	1.73	0.084
**Trait mindfulness -acceptance (actor)**	0.25	0.13	0	0.5	91	2.02	0.047
**Trait mindfulness -attention (actor)**	0.51	0.13	0.25	0.78	91	3.86	<.001
**State mindfulness - acceptance (partner)**	-0.06	0.07	-0.19	0.07	1042	-0.93	0.352
**State mindfulness - attention (partner)**	-0.02	0.06	-0.13	0.09	1042	-0.35	0.730
**Trait mindfulness**	-0.32	0.12	-0.56	-0.07	91	-2.59	0.011

**- acceptance (partner)**							
**Trait mindfulness – attention (partner)**	0.26	0.13	0	0.52	91	2.01	0.048
**Day of survey**	-0.07	0.01	-0.1	-0.05	1042	-5.91	<.001

Interaction of Attention and Acceptance. In line with Monitor and Acceptance Theory (Lindsay & Creswell, 2017) we exploratorily tested interactions between mindful attention and acceptance but did not find support for this. Results can be found in Table S11.

Table S11

Hypothesis 1: Partner Ratings of Compassion

Predictor	Estimate	SE	95% CI lower	95% CI upper	df	t	p
**Intercept**	6.72	5.15	-3.37	16.82	1042	1.31	0.192
**State mindfulness - acceptance (actor)**	0.22	0.31	-0.39	0.83	1042	0.71	0.475
**State mindfulness - attention (actor)**	0.37	0.31	-0.23	0.97	1042	1.2	0.231
**Trait mindfulness -acceptance (actor)**	-0.27	0.74	-1.74	1.2	86	-0.37	0.715
**Trait mindfulness -attention (actor)**	0.34	0.64	-0.93	1.61	86	0.53	0.597
**State mindfulness - acceptance (partner)**	-0.23	0.33	-0.88	0.43	1042	-0.68	0.497
**State mindfulness - attention (partner)**	-0.62	0.29	-1.19	-0.04	1042	-2.1	0.036
**Trait mindfulness - acceptance (partner)**	-0.53	0.76	-2.05	0.98	86	-0.7	0.486
**Trait mindfulness – attention (partner)**	0.12	0.68	-1.22	1.47	86	0.18	0.855
**State mindfulness acc X State mindfulness att (actor)**	-0.05	0.04	-0.14	0.03	1042	-1.24	0.216
**Trait mindfulness acc X Trait mindfulness att (actor)**	0.04	0.15	-0.25	0.34	86	0.3	0.767
**State mindfulness acc X Trait mindfulness att (actor)**	-0.01	0.07	-0.14	0.12	1042	-0.12	0.905

**State mindfulness att X Trait mindfulness acc (actor)**	-0.08	0.07	-0.22	0.07	1042	-1.07	0.286
**State mindfulness acc X State mindfulness att (partner)**	0.03	0.04	-0.05	0.12	1042	0.83	0.409
**Trait mindfulness acc X Trait mindfulness att (partner)**	0.09	0.16	-0.22	0.4	86	0.57	0.572
**State mindfulness acc X Trait mindfulness att (partner)**	0.12	0.07	-0.02	0.27	1042	1.65	0.098
**State mindfulness att X Trait mindfulness acc (partner)**	0.13	0.07	-0.01	0.27	1042	1.87	0.062

Investigation of Ceiling Effects. We investigated whether there were ceiling effects for between-person mindfulness, but did not find evidence for this, as 7.4% of participants scored at the maximum possible value for trait mindful attention and 4.8% of participants scored at the maximum possible value for trait mindful acceptance, which are both below the conventional 15% cutoff (Terwee et al., 2007).

Supplementary Note 2: Trait Analyses and Results

Our trait data had a two-level structure, with participants nested within dyads. We used a multilevel model with random intercepts per dyad. We conducted the same model comparisons as with the daily diary data and likewise selected non-distinguishable APIM models (model selection reported in Table S12).

Model Selection

Table S12a

Model Selection for Hypothesis 1: Partner Ratings of Compassion

Model	df	AIC	BIC	logLik	Test	L.Ratio	p-value
**APIM-Distinguishable**	8	1491.143	1517.161	-737.5714			
**APIM-Indistinguishable**	**5**	**1491.835**	**1508.097**	**-740.9177**	**1 vs 2**	**6.692616**	**0.0824**
**Non-APIM**	4	1498.049	1511.059	-745.0247	2 vs 3	8.213988	0.0042

Table S12b

Model Selection for Hypothesis 1: Self Ratings of Compassion

Model	df	AIC	BIC	logLik	Test	L.Ratio	p-value
**APIM-Distinguishable**	8	1371.544	1397.562	-677.7718			
**APIM-Indistinguishable**	**5**	**1367.630**	**1383.891**	**-678.8150**	**1 vs 2**	**2.086321**	**0.5547**
**Non-APIM**	4	1370.297	1383.306	-681.1486	2 vs 3	4.667117	0.0307

Table S12c

Model Selection for Hypothesis 2: Path a

Model	df	AIC	BIC	logLik	Test	L.Ratio	p-value
**APIM-Distinguishable**	8	1428.245	1454.305	-706.1224			
**APIM-Indistinguishable**	**5**	**1428.087**	**1444.375**	**-709.0436**	**1 vs 2**	**5.842459**	**0.1195**
**Non-APIM**	4	1426.146	1439.176	-709.0729	2 vs 3	0.058547	0.8088

Table S12d

Model Selection for Hypothesis 2: Full Model, Partner Ratings of Compassion

Model	df	AIC	BIC	logLik	Test	L.Ratio	p-value
**APIM-Distinguishable**	12	1492.101	1531.128	-734.0505			
**APIM-Indistinguishable**	**7**	**1489.357**	**1512.123**	**-737.6785**	**1 vs 2**	**7.255855**	**0.2023**
**Non-APIM**	5	1500.049	1516.310	-745.0245	2 vs 3	14.692124	0.0006

Table S12e

Model Selection for Hypothesis 2: Full Model, Self Ratings of Compassion

Model	df	AIC	BIC	logLik	Test	L.Ratio	p-value
**APIM-Distinguishable**	12	1374.486	1413.513	-675.2430			

**APIM-Indistinguishable**	**7**	**1367.500**	**1390.266**	**-676.7500**	**1 vs 2**	**3.013983**	**0.6978**
**Non-APIM**	5	1372.254	1388.515	-681.1271	2 vs 3	8.754193	0.0126

Table S12f

Model Selection for Hypothesis 3: Partner Ratings of Compassion

Model	df	AIC	BIC	logLik	Test	L.Ratio	p-value
**APIM-Distinguishable**	20	1376.536	1440.171	-668.2678			
**APIM-Indistinguishable**	**11**	**1372.458**	**1407.458**	**-675.2290**	**1 vs 2**	**13.92253**	**0.1251**
**Non-APIM**	7	1394.059	1416.331	-690.0293	2 vs 3	29.60055	<.0001

Table S12g

Model Selection for Hypothesis 3: Self Ratings of Compassion

Model	df	AIC	BIC	logLik	Test	L.Ratio	p-value
**APIM-Distinguishable**	20	1253.888	1317.524	-606.9442			
**APIM-Indistinguishable**	**11**	**1242.891**	**1277.890**	**-610.4454**	**1 vs 2**	**7.002377**	**0.6369**
**Non-APIM**	7	1249.192	1271.465	-617.5960	2 vs 3	14.301277	0.0064

Results

Table S13

Descriptive Statistics and Correlations

	M	SD (BD)	SD (WD)	1	2	3	4	5
**Mindfulness (1)**	6.27	1.27	0.83		-0.59***	-0.07	-0.08	0.10
**Self-absorption (2)**	3.66	1.30	1.03	-0.62***		0.17*	0.17*	0.04

**Self-construal (3)**	7.71	1.04	0.65	0.08	-0.11		-0.03	0.27***
**Compassion-partner-rated (4)**	7.45	1.50	0.91	0.29**	-0.27**	0.27**		-0.08
**Compassion-self-rated (5)**	8.19	1.08	0.64	0.34***	-0.33***	0.36***	0.69***	

Note: Means and standard deviations rescaled to 1-10 for comparability. SD (BD) is between-dyad standard deviation, SD (WD) is within-dyad standard deviation. Upper triangle shows within-dyad correlations; lower triangle shows between-dyad correlations with significance indicated by asterisks (* p<.05, ** p<.01, *** p<.001).

Hypothesis 1: Mindfulness is positively related to compassion

We found support for this hypothesis using self ratings of compassion (t(91)=3.32, B=.34, SE=.10, p=.001, 95% CI[0.14, 0.54]) but only marginal support using partner ratings of compassion (t(91)=1.75, *B*=.25, SE=.14, p=.084, 95% CI[-0.03, 0.53]). See Table S14 for full results.

Table S14a

Hypothesis 1: Partner Ratings of Compassion

Effect	Estimate	SE	t	df	95% CI lower	95% CI upper	p
**Intercept**	37.87	6.54	5.79	111	25	50.74	<.001
**Mindfulness (actor)**	0.25	0.14	1.75	91	-0.03	0.53	0.084
**Mindfulness (partner)**	0.41	0.14	2.89	91	0.13	0.69	0.005

Note: p values reported exactly to three decimals; p < .001 when smaller. Actor = participant’s own state/trait; Partner = partner’s concurrent state/trait.

Table S14b

Hypothesis 1: Self Ratings of Compassion

Effect	Estimate	SE	t	df	95% CI lower	95% CI upper	p
**Intercept**	47.31	4.71	10.04	111	38.04	56.58	<.001
**Mindfulness (actor)**	0.34	0.10	3.32	91	0.14	0.54	0.001
**Mindfulness (partner)**	0.20	0.10	1.95	91	0	0.4	0.054

Note: p values reported exactly to three decimals; p < .001 when smaller. Actor = participant's own state/trait; Partner = partner's concurrent state/trait.

Hypothesis 2: The relationship between mindfulness and compassion is mediated by self-absorption

Regarding our mediation hypothesis, mindfulness did predict self-absorption (t(92)=-10.6, *B*=-1.30, SE=.12, p<.001, 95% CI[-1.54, -1.06]), but self-absorption did not predict partner-rated compassion (t(89)= -.12, *B*=-.01, SE=.08, p=.902, 95% CI[-.17, 0.15]), nor self-rated compassion (t(89)= -.84, *B*=-.05, SE=.06, p=.405, 95% CI[-.17, .07]). Therefore, we did not find support for our mediation hypothesis. See Table S15 for full results.

Table S15a

Hypothesis 2: Path a

Effect	Estimate	SE	t	df	95% CI lower	95% CI upper	p
**Intercept**	77.42	4.88	15.86	111	67.75	87.09	<.001
**Mindfulness (actor)**	-1.3	0.12	-10.6	92	-1.54	-1.06	<.001
**Mindfulness (partner)**	0.08	0.12	0.67	92	-0.16	0.32	0.507

Note: p values reported exactly to three decimals; p < .001 when smaller. Actor = participant's own state/trait; Partner = partner's concurrent state/trait.

Table S15b

Hypothesis 2: Full Model, Partner Ratings of Compassion

Effect	Estimate	SE	t	df	95% CI lower	95% CI upper	p
**Intercept**	52.23	11.76	4.44	111	29.21	75.25	<.001
**Mindfulness (actor)**	0.25	0.18	1.41	89	-0.1	0.60	0.162

**Mindfulness (partner)**	0.19	0.18	1.04	89	-0.16	0.53	0.300
**Self-absorption (actor)**	-0.01	0.08	-0.12	89	-0.17	0.15	0.902
**Self-absorption (partner)**	-0.18	0.08	-2.11	89	-0.34	-0.01	0.038

Note: p values reported exactly to three decimals; p < .001 when smaller. Actor = participant's own state/trait; Partner = partner's concurrent state/trait.

Table S15c

Hypothesis 2: Full Model, Self Ratings of Compassion

Effect	Estimate	SE	t	df	95% CI lower	95% CI upper	p
**Intercept**	61.38	8.4	7.31	111	44.94	77.82	<.001
**Mindfulness (actor)**	0.28	0.13	2.25	89	0.04	0.53	0.027
**Mindfulness (partner)**	0.03	0.13	0.24	89	-0.22	0.28	0.812
**Self-absorption (actor)**	-0.05	0.06	-0.84	89	-0.17	0.07	0.405
**Self-absorption (partner)**	-0.13	0.06	-2.23	89	-0.25	-0.02	0.028

Note: p values reported exactly to three decimals; p < .001 when smaller. Actor = participant's own state/trait; Partner = partner's concurrent state/trait.

Hypothesis 3: The mediation of the relationship between mindfulness and compassion by self-absorption is moderated by self-construal

We did not find support for this hypothesis, as there was no significant interaction between self-absorption and interdependent self-construal for either partner ratings (t(85)=0.94, *B*=.01, SE=.01, p=.351, 95% CI[-0.01, 0.03]) nor for self ratings (t(85)=1.17, *B*=.01, SE=.01, p=.245, 95% CI[-0.01, 0.02]) of compassion. See Table S16 for full results.

Table S16a

Hypothesis 3: Partner Ratings of Compassion

Effect	Estimate	SE	t	df	95% CI lower	95% CI upper	p
**Intercept**	40.89	30.84	1.33	93	-18.85	100.64	0.188
**Mindfulness (actor)**	0.27	0.18	1.5	85	-0.08	0.62	0.138
**Mindfulness (partner)**	0.19	0.18	1.07	85	-0.16	0.54	0.288
**Self-absorption (actor)**	-0.47	0.49	-0.97	85	-1.42	0.48	0.336
**Self-construal (actor)**	-0.04	0.37	-0.1	85	-0.75	0.67	0.921
**Self-absorption (partner)**	-0.39	0.49	-0.8	85	-1.34	0.56	0.424
**Self-construal (partner)**	0.27	0.37	0.73	85	-0.44	0.98	0.466
**Self-absorption x self-construal (actor)**	0.01	0.01	0.94	85	-0.01	0.03	0.351
**Self-absorption x self-construal (partner)**	0	0.01	0.4	85	-0.01	0.02	0.689

Note: p values reported exactly to three decimals; p < .001 when smaller. Actor = participant’s own state/trait; Partner = partner’s concurrent state/trait.

Table S16b

Hypothesis 3: Self Ratings of Compassion

Effect	Estimate	SE	t	df	95% CI lower	95% CI upper	p
**Intercept**	55.43	21.16	2.62	93	14.43	96.43	0.01
**Mindfulness (actor)**	0.25	0.12	2	85	0.01	0.49	0.049
**Mindfulness (partner)**	0.01	0.12	0.05	85	-0.23	0.25	0.959
**Self-absorption (actor)**	-0.47	0.34	-1.4	85	-1.12	0.18	0.165
**Self-construal (actor)**	0.19	0.25	0.77	85	-0.3	0.68	0.446
**Self-absorption (partner)**	-0.34	0.34	-1.02	85	-1	0.31	0.309
**Self-construal (partner)**	-0.01	0.25	-0.02	85	-0.49	0.48	0.983

**Self-absorption x self-construal (actor)**	0.01	0.01	1.17	85	-0.01	0.02	0.245
**Self-absorption x self-construal (partner)**	0	0.01	0.63	85	-0.01	0.02	0.531

Discussion

The results from the trait data showed some differences from the results from the daily diary data. We found some support for the hypothesis that mindfulness is related to compassion, but this was only marginal in the case of partner-rated compassion. Though we found mindfulness to predict self-absorption, we did not find an association between self-absorption and compassion, and therefore did not find a mediation in the trait data, though we did in the daily diary data. Similar to the daily diary data, we did not find a moderation by self-construal. It is important to note that we conducted our power analysis in terms of our daily diary data. Since repeated measures data is better powered, the trait analyses reported here may have been underpowered. Further, there may be differences resulting from possible recall bias introduced via the use of the trait measurements. Therefore, these results should be interpreted with caution.

Supplementary References

Lindsay, E. K., & Creswell, J. D. (2017). Mechanisms of mindfulness training: Monitor and Acceptance Theory (MAT). *Clinical Psychology Review*, *51*, 48–59. https://doi.org/10.1016/j.cpr.2016.10.011

Terwee, C. B., Bot, S. D. M., De Boer, M. R., Van Der Windt, D. A. W. M., Knol, D. L., Dekker, J., Bouter, L. M., & De Vet, H. C. W. (2007). Quality criteria were proposed for measurement properties of health status questionnaires. *Journal of Clinical Epidemiology*, *60*(1), 34–42. https://doi.org/10.1016/j.jclinepi.2006.03.012
